# Gambling Disorder and Increased Psychiatric Comorbidity: A Finnish Register-Based Study

**DOI:** 10.1177/14550725251380172

**Published:** 2025-09-30

**Authors:** Anne H. Salonen, Tiina A. Latvala, Miika Vuori, Jonna Levola, Sari Castrén, Tanja Grönroos

**Affiliations:** 1 3837 Finnish Institute for Health and Welfare, Department of Healthcare and Social Welfare, Helsinki, Finland; 2 University of Eastern Finland, Faculty of Health Sciences, Kuopio, Finland; 3 Kela Research, 8054 the Social Insurance Institution of Finland, Helsinki, Finland; 4 3835 University of Helsinki, Department of Psychiatry, Helsinki, Finland; 5 Psychiatry, Hospital District of Helsinki and Uusimaa, HUS, Finland; 6 Social Sciences Department of Psychology and Speech-Language Pathology Turku, University of Turku, Turku, Finland; 7 Department of Medicine, 3835 University of Helsinki, Helsinki, Finland; 8 University of Helsinki, Faculty of Social Sciences, Helsinki, Finland

**Keywords:** comorbid disorder, comorbidities, gambling disorder, gender-specific, ICD-10, incidence, pathological gambling, psychiatric disorder, register-based population study

## Abstract

**Aim:**

This study investigates gender-specific standardized incidence ratios (SIRs) of comorbid psychiatric disorders among adults diagnosed with gambling disorder (GD) and also examines mortality rates and causes of death in this population.

**Methods:**

The study included all individuals aged 18 years or older in Finland diagnosed with GD between 2011 and 2022 (*n* = 3,605), as defined by ICD-10 code F63.0 (International Classification of Diseases, 10th Revision). Data were drawn from nationwide social and healthcare registers, covering both primary and specialized care. The general population (*n* = 4,374,192) served as the reference group.

**Results:**

Among individuals with GD, 88.5% were diagnosed with at least one additional psychiatric disorder. After age standardization, the incidence of psychiatric comorbidities was significantly higher in the GD group compared to the general population. Mood and anxiety disorders were the most common disorders in both groups. However, personality disorders (PD), schizophrenia spectrum disorders (SSD) and substance use disorders (SUD) were significantly more common among individuals with GD. Some gender-specific patterns emerged: GD was particularly associated with PD and SUD among women, as well as with PD and SSD among men. Of the GD cohort, 3.6% had died, with suicide accounting for 22% of deaths.

**Conclusions:**

Diagnosed GD is associated with elevated rates of psychiatric disorders, particularly PD, SSD and SUD. These findings highlight the importance of comprehensive mental health assessment in individuals with GD. Frontline social and healthcare professionals should be attentive to the high occurrence of psychiatric comorbidities in this population to ensure timely and appropriate care.

## Introduction

Based on a meta-analysis including general population samples from 68 countries and territories, 1.4% (range 1.3–2.4%) of the adults were engaging in problem gambling and 8.7% in at-risk gambling (Tran et al., 2024). The terms at-risk and problem gambling are often used in community and population-based surveys (Calado & Griffiths, 2016; Gabellini et al., 2023). Problem gambling refers to an intermediate or subclinical form of the gambling disorder (GD), while GD is defined as gambling that is severe enough to fulfil diagnostic criteria. GD is a behavioural addiction with similar aetiology and symptoms to those of substance use disorders (SUD) (APA, 2013; Grant et al., 2010). GD corresponds to pathological gambling (International Classification of Diseases, 10th Revision, code F63.0). Based on both population-based surveys and clinical data, psychiatric comorbidity is related to both GD and problem gambling (Cowlishaw et al., 2014; Dowling et al., 2015; Lorains et al., 2011; Rash et al., 2016; Richard et al., 2020; Shaffer & Korn, 2002). To our knowledge, the administrative incidence rate of the GD (clinically diagnosed and recorded cases according to healthcare data) in the total population or its comorbidities has been studied only in Nordic register-based studies.

### Psychiatric Comorbidity in Individuals with Problem Gambling and GD

Psychiatric comorbidity is strongly associated with problem gambling and GD. A recently published register study showed that of all somatic illnesses and psychiatric disorders, the most common comorbid conditions among people with GD in Finland included psychiatric disorders (Grönroos et al., 2024). The prevalence of psychiatric disorders, excluding some of the organic memory disorders, was 87%. Corresponding rates in Swedish register study were lower, since, in Sweden, 73% of persons with diagnosed GD had at least one comorbid psychiatric diagnosis (Håkansson et al., 2018). However, previous Swedish and Norwegian registers have covered only specialized health care (Håkansson et al., 2018; Larsson & Håkansson, 2022; Leino et al., 2023), while the Finnish studies also covered primary health care and social services (Grönroos et al., 2024; Salonen et al., 2022).

In register studies, population surveys and clinical studies, mood disorders, anxiety disorders and SUD are consistently identified as the most common psychiatric comorbidities in individuals with problem gambling and GD. A meta-analysis of population surveys indicate that the highest mean prevalence was for nicotine dependence (60.1%), SUD (57.5%), mood disorders (37.9%) and anxiety disorders (37.4%) (Lorains et al., 2011). Based on a Norwegian register-based study, one in five (22.5%) of people with GD in Norway were diagnosed with a SUD, which include excessive use of alcohol, drugs, nicotine and/or other psychoactive substances (Leino et al., 2023). Conversely, 0.7% of individuals with SUD were also diagnosed with GD. Another review also discovered that there were high rates of current mood disorders (23.1%), alcohol use disorders (AUD) (21.2%), anxiety disorders (17.6%) and non-alcohol related SUD (7.0%) among treatment-seeking people with gambling problems (Dowling et al., 2015). In addition, the highest mean prevalence rate was observed for nicotine dependence (56.4%) and major depressive disorder (29.9%), and smaller estimates for alcohol abuse (18.2%), alcohol dependence (15.2%) and cannabis use disorder (11.5%). It was further estimated that around 14% of patients in substance use treatment had comorbid GD and 23% suffered conditions along the broader spectrum of problem gambling (Cowlishaw et al., 2014).

Based on a meta-analysis, the following disorders have also been identified among people seeking treatment for gambling problems: social anxiety disorder (14.9%), generalized anxiety disorder (14.4%), panic disorder (13.7%), post-traumatic stress disorder (PTSD; 12.3%), attention-deficit hyperactivity disorder (ADHD; 9.3%), adjustment disorder (9.2%), bipolar disorder (8.8%) and obsessive-compulsive disorder (8.2%) (Dowling et al., 2015). Furthermore, comorbid antisocial personality disorder has been linked with GD (Blum et al., 2017). Yet, this group represents a minority when compared with those people who have acquired their antisocial traits because of GD (Crockford & el-Guebaly, 1998).

Additionally, a variety of stressful and traumatic life events across the life span are associated with developing GD (Moore & Grubbs, 2021). The impact of PTSD is highest, and experienced trauma contributes to comorbid mood, anxiety and SUD. Problem gambling may also be used to regulate mood (i.e., escape) and serve as a coping to relieving emotional stress thus may aggravate the symptomology of GD (Kruger et al., 2020; Matheson et al., 2018).

### Deaths and Causes of Death Among Individuals with GD

Swedish register-based studies have observed that mortality and suicide death rates are significantly elevated among people with GD (Håkansson & Karlsson, 2020; Karlsson & Håkansson, 2018). The outcome measure of their study was defined as a suicide attempt or death from suicide. Based on their results, suicidal behaviour was linked with female gender, mood disorders, anxiety disorders and SUD (Håkansson et al., 2020). Review studies have found a positive association between suicide attempts, suicidal deaths and self-harm among gamblers (Andreeva et al., 2022; Armoon et al., 2023; Gray et al., 2021; Kristensen et al., 2024; Pfuhlmann & Schmidtke, 2002). Gambling and suicidal behaviour are linked to indebtedness and shame and is also related to psychiatric conditions, chronic physical illnesses, personality traits and life conditions (Armoon et al., 2023; Marionneau & Nikkinen, 2022; van der Maas et al., 2024).

### Gender Differences in Comorbidities Among Individuals with Problem Gambling and GD

Previous research strongly indicates that GD and problem gambling are more frequent among men than women (Hing et al., 2016; Sharman et al., 2019). The Swedish register study found that 77% of people with GD diagnoses were men, while the corresponding figure was 66% in the Finnish study (Håkansson et al., 2018; Salonen et al., 2022). However, several register-based studies indicate that psychiatric comorbidity is more common among women with diagnosed GD compared to men (Håkansson et al., 2018; Larsson & Håkansson, 2022). In Sweden, the prevalence rates of comorbid psychiatric diagnoses in people with GD were more common among women (77%) than among men (71%) (Håkansson et al., 2018). Furthermore, many diagnostic subgroups were more common among women, with anxiety and affective disorders being the most prevalent ones. Nevertheless, SUD were equally prevalent among both genders with GD, while SUD is clearly more prevalent among men in the general population. Norwegian researchers also identified sex differences in the risk trajectories of the other addictive disorder over time between individuals with GD and SUD: compared with men with GD, the risk of SUD over time was greater among women with GD (Leino et al., 2023).

In addition to the differences described above, there are also distinctions between women and men in gambling behaviour and the development of GD (Abbott et al., 2018). Women typically begin gambling at an older age than men; however, they tend to develop gambling problems more rapidly once they start, a phenomenon known as the telescoping effect (Miller et al., 2023; Syvertsen et al., 2023). Furthermore, women seek help for gambling problems earlier than men. They may also be more likely to use gambling as a coping mechanism for depression and anxiety, which can lead to excessive gambling (Desai & Potenza, 2008). For these reasons, we will examine women and men separately in this study.

Despite the vast body of published literature on GD and comorbid disorders, previous studies on the topic covering the general population, particularly from the perspective of both primary and specialized care, are rather scarce. This study focuses on people with GD diagnoses based on comprehensive Finnish national health registers, among both men and women, and examines: (1) comorbid diagnoses of psychiatric disorders and (2) death rates and causes of death in the context of primary and specialized health care. The corresponding standardized incidence rates (SIR) in the general population for comorbid psychiatric disorders are presented for reference.

## Methods

Diagnoses examined in this paper are based on the International Classification of Diseases, 10th Revision (ICD-10) criteria. For the sake of clarity, the ICD-10 code F63.0 refers to pathological gambling. However, the more recent term GD is used in this paper due to the changes in the revised edition of the (11th edition; ICD-11; World Health Organization, 2019). In the ICD-11, the diagnostic term pathological gambling was changed to GD and placed under the category Disorders due to substance use or addictive behaviours.

### Participants and Procedures

Diagnoses of GD and other psychiatric disorders were retrieved from the national registers administrated by the Finnish Institute for Health and Welfare (THL). These registers included: Register of Primary Health Care visits, and Care Register for Health Care, including specialized outpatient and inpatient health care, as well as inpatient social care. The register of Primary Health Care Visits covers all primary healthcare centres in Finland. Health visits at occupational healthcare services and private clinics are also included. The Care Register for Health Care contains data on specialized outpatient and inpatient care including information in regard to each visit, such as diagnoses. The Care Register for Social Welfare covers registers of social welfare institutions, such as nursing homes, disability homes, inpatient substance use care and other 24/7 sheltered accommodation.

Aggregated data including primary, secondary and the long-term diagnoses were created as follows. First, people with GD (ICD-10; F63.0) diagnosed between 2011 and 2022 were selected. Then, comorbid psychiatric disorders (F00–99, excluding F63.0) diagnosed before, at the same time or after the diagnosis of GD (between 2011 to 2022) were included in the analyses. Each person and each of their diagnosis were counted only once. In addition, information about death rates and causes of death were retrieved.

Exclusion criteria included underaged individuals (*n* = 108) and individuals living outside Mainland Finland (*n* = 8). Ultimately, a total of 3,605 individuals living in Mainland Finland with registered GD diagnoses were included. The age of individuals with GD diagnoses varied from 18 to >90 years (mean age = 35.4 years). A reference group including the average population (4,374,192) with the same age range (mean age = 50.4 years) was retrieved from the same national registry. Due to age differences between individuals with GD and the general population, we used estimates of disorder incidence relatively to what would be expected if individuals with GD had the same morbidity profile as the general population. Both study populations were stratified by gender.

### Statistical Analysis

Data were processed and analysed using Excel (Microsoft Corp.) and R epitools package (Anderson & Rosenberg, 1998; Fay & Feuer, 1997). First, the results are presented using descriptive statistics, such as percentages and frequencies. The incidences of psychiatric disorders (i.e., first recorded ICD-10 F code) were calculated separately for women and men with diagnosed GD and for the general population. Furthermore, the expected number of psychiatric disorder cases was calculated by summing the products of the age-specific expected case numbers. These were calculated by multiplying each age-specific population of the people with GD by corresponding age-specific incidence rate of the general population. The observed number of cases with a diagnosis of a psychiatric disorder was divided by the expected number of cases, and the results was multiplied by 100 to calculate SIRs (Jensen et al., 1991). In the present study, SIR is an estimate of the observed number of incident cases with comorbid psychiatric disorder diagnoses among GD population when compared with total population (without GD) average. The interpretation of the SIR is as follows: if the SIR is 100, this means that the observed number of cases among people with GD is equal to the expected number of cases if people with GD had the same disorder rate as the general population. Similarly, an SIR of 125 means that there are 25% more cases than expected, while an SIR of 75 indicates that there are 25% fewer cases than expected. The 95% confidence interval (CI) for the SIR was calculated using a short-cut method (Jensen et al., 1991).

## Results

### Incidence of Comorbid Psychiatric Illnesses Among Individuals with GD

Of individuals diagnosed with GD, 88.5% had been diagnosed with at least one additional psychiatric disorder (Table 1). The most common comorbid psychiatric disorders were mood disorders (65.5%), anxiety disorders (58.7%) and SUD (32.4%). Unipolar depressive disorders/episodes (61.1%) were the most common types of mood disorders. Correspondingly, phobic anxiety disorders (48.8%) and adjustment and stress related disorders (21.6%) were the most common type of anxiety disorder, while AUD (24.2%) were the most common SUD. Both women and men with GD had the same three most common comorbid psychiatric disorders (Tables 2 and 3). However, among women, personality disorders (26.8%) and behavioural disorders associated with psychological disturbances and physical factors (26.7%) were almost as common as SUD (30.7%).

**Table 1.** Observed (Obs) and Expected (Exp) Number of Cases of Psychiatric Disorders and Standardized Incidence Ratios (SIRs) with 95% Confidence Intervals (95% CI) Among Persons with Diagnosed Gambling Disorder (GD; F63.0) in 2011–2022 (Reference Population: Finnish General Population).

		Finnish population (N = 4,374,192)		Persons with GD (N = 3,605)				
	Diagnostic group	%	*N*	%	*N* observed	*N* expected	SIR	95% CI
**F00–99 excluding F63.0**	**Mental disorder**	37.2	1,626,457	88.5	3,189	1,492	213.7	206.3–221.2
**F00–09**	**Organic mental disorder**	5.6	246,823	2.6	95	31	304.3	246.2–368.6
**F10–19**	**Substance use disorder (SUD)**	6.0	262,356	32.4	1,167	229	510.3	481.4–540.0
F10	Alcohol use disorder (AUD)	4.6	201,217	24.2	873	164	530.7	496.1–566.5
F11	Opioid use disorders (OUD)	0.4	17,999	2.4	87	25	343.9	275.4–419.9
F12	Cannabis use disorder (CUD)	0.3	14,028	4.5	163	23	723.5	616.7–838.9
F13	Benzodiazepine/hypnotics use disorder	0.6	24,165	4.9	176	26	674.5	578.6–777.9
F14–15	Cocaine/other stimulant use disorder	0.2	10,094	2.2	79	16	504.5	399.4–621.9
F16, F18–19	Other or multiple drug use disorder	0.6	27,365	6.8	246	40	618.4	543.5–698.1
F17	Tobacco use disorder	0.8	34,846	3.6	130	25	519.5	434.0–612.6
**F20–29**	**Schizophrenia spectrum disorder**	2.4	104,071	13.6	492	93	530.9	485.0–578.8
F20	Schizophrenia	1.0	43,337	5.0	181	36	506.0	435.0–582.4
F21	Schizotypal disorder	0.1	4,152	0.6	23	5	490.7	310.7–711.8
F22	Persistent delusional disorder	0.5	20,428	1.8	64	10	632.6	487.1–797.0
F23	Acute psychosis	0.4	15,692	2.5	91	17	525.0	422.6–638.3
F25	Schizoaffective disorders	0.3	12,946	2.9	105	12	852.4	697.1–1023.2
F28–29	Other/unspecified non-organic psychosis	1.1	49,165	8.9	320	57	562.8	502.8–626.2
**F30–39**	**Mood disorders**	13.9	609,998	65.5	2,361	663	355.9	341.7–370.4
F30–31	Bipolar disorders	1.3	58,050	12.9	466	64	729.4	664.7–797.1
F32–34	Unipolar depression including dysthymia	13.2	576,719	61.1	2,201	630	349.6	335.2–364.4
F38–39	Other/unspecified mood disorder	0.6	27,942	5.3	192	36	538.0	464.6–616.7
**F40–48**	**Anxiety disorders**	18.3	799,014	58.7	2,117	932	227.2	217.6–237.0
F40–41	Phobic anxiety disorders	11.5	505,079	48.8	1,758	628	279.8	266.9–293.0
F42	Obsessive-compulsive disorder	0.7	29,529	4.6	165	43	380.1	324.3–440.3
F43–44	Adjustment and stress related disorders	7.1	312,629	21.6	778	346	225.0	209.4–241.0
F45	Somatoform disorders	3.0	132,726	4.9	176	142	123.5	106.0–142.5
**F50–59**	**Behavioural syndromes associated with psychological disturbances and physical factors**	9.3	406,300	22.7	820	376	218.1	203.5–233.3
F50	Eating disorders	0.5	21,141	1.5	53	32	163.6	122.5–210.6
F51	Non-organic sleep disorders	8.2	358,316	19.9	719	319	225.1	209.0–241.9
F52	Sexual dysfunction	0.7	31,879	2.0	72	30	237.8	186.1–296.0
**F60–69 excluding F63.0**	**Personality disorders**	1.8	79,158	17.9	645	105	616.6	570.0–665.1
**F70–79**	**Intellectual disability**	0.5	23,490	1.1	41	26	158.2	113.5–210.3
**F80–89**	**Disorders of psychological development**	0.8	36,562	4.2	153	52	295.5	250.5–344.2
F80–83	Other developmental disorders	0.5	22,306	2.6	95	30	320.0	258.9–387.6
F84, excluding F84.2–84.4	Autism spectrum disorders	0.4	15,434	1.7	63	24	260.7	200.3–329.1
**F90–98**	**Behavioural and emotional disorders with onset usually occurring in childhood and adolescence**	2.0	85,583	12.3	445	128	348.2	316.6–381.3
F90.0, F90.8, F90.9, F98.8	ADHD spectrum	1.6	69,806	10.5	380	107	354.5	319.7–391.0
F91–F92, F90.1	Conduct disorder	0.1	4,687	1.1	41	6	646.5	463.8–859.6
**F99**	**Unspecified mental disorder**	0.5	23,477	4.6	167	28	591.8	505.4–685.0

Bolded terms indicate diagnostic categories under which specific diagnoses are listed.

**Table 2.** Observed (Obs) and Expected (Exp) Number of Cases of Psychiatric Disorders and Standardized Incidence Ratios (SIRs) with 95% Confidence Intervals (95% CI) Among Men with Diagnosed Gambling Disorder (GD; F63.0) in 2011–2022 (Reference Population: Men in Finnish General Population).

		Men in Finnish population (*N* = 2,142,986)		Men with GD (*N* = 2,574)				
	Diagnostic group	%	*N*	%	*N* observed	*N* expected	SIR	95% CI
**F00–99 excluding F63.0**	**Mental disorder**	32.1	687,783	87.0	2,240	911	245.8	235.7–256.1
**F00–09**	**Organic mental disorder**	4.9	105,984	2.2	56	19	300.8	227.2–384.8
**F10–19**	**Substance use disorder (SUD)**	8.3	177,592	33.0	850	214	397.8	371.5–425.0
F10	Alcohol use disorder (AUD)	6.6	142,332	25.2	649	156	417.2	385.7–449.9
F11	Opioid use disorders (OUD)	0.6	12,016	2.2	57	25	231.0	174.9–294.9
F12	Cannabis use disorder (CUD)	0.5	10,987	5.4	138	27	512.6	430.6–601.7
F13	Benzodiazepine/hypnotics use disorder	0.7	14,524	4.5	115	25	463.1	382.3–551.6
F14–15	Cocaine/other stimulant use disorder	0.3	7,101	2.5	64	16	395.1	304.2–497.8
F16, F18–19	Other or multiple drug use disorder	0.9	18,763	7.4	191	41	464.2	400.7–532.4
F17	Tobacco use disorder	0.9	19,162	2.6	68	17	398.8	309.7–499.3
**F20–29**	**Schizophrenia spectrum disorder**	2.4	51,623	14.6	376	76	491.6	443.2–542.6
F20	Schizophrenia	1.1	23,680	5.8	149	31	473.2	400.3–552.2
F21	Schizotypal disorder	0.1	2,405	0.6	15	4	358.7	200.1–563.2
F22	Persistent delusional disorder	0.4	7,709	1.8	47	8	606.4	445.4–792.1
F23	Acute psychosis	0.4	7,913	3.0	77	14	537.1	423.9–663.8
F25	Schizoaffective disorders	0.3	5,503	2.7	70	8	880.6	686.4–1098.9
F28–29	Other/unspecified non-organic psychosis	1.2	25,699	9.7	249	49	512.2	450.6–577.8
**F30–39**	**Mood disorders**	10.7	228,736	61.2	1,575	367	429.7	408.7–451.1
F30–31	Bipolar disorders	1.2	24,755	11.0	283	37	765.3	678.7–857.1
F32–34	Unipolar depression including dysthymia	9.9	212,979	56.7	1,459	344	424.5	403.0–446.5
F38–39	Other/unspecified mood disorder	0.5	11,443	4.9	127	21	590.8	492.5–698.0
**F40–48**	**Anxiety disorders**	12.7	272,907	54.4	1,401	481	291.2	276.1–306.6
F40–41	Phobic anxiety disorders	8.3	178,538	45.1	1,161	336	345.1	325.5–365.2
F42	Obsessive-compulsive disorder	0.5	11,413	3.8	97	25	394.0	319.5–476.4
F43–44	Adjustment and stress related disorders	4.3	92,304	17.4	447	152	294.1	267.5–322.0
F45	Somatoform disorders	1.7	37,457	4.4	113	55	204.3	168.4–243.7
**F50–59**	**Behavioural syndromes associated with psychological disturbances and physical factors**	7.7	164,755	21.2	545	217	251.6	230.9–273.2
F50	Eating disorders	0.1	1,723	0.5	13	3	376.2	199.5–608.5
F51	Non-organic sleep disorders	6.4	136,530	18.7	481	180	267.5	244.1–291.9
F52	Sexual dysfunction	1.4	30,156	2.7	70	39	181.8	141.7–226.9
**F60–69 excluding F63.0**	**Personality disorders**	1.4	29,492	14.3	369	54	678.6	611.2–749.6
**F70–79**	**Intellectual disability**	0.6	13,014	1.1	28	22	126.9	84.3–178.3
**F80–89**	**Disorders of psychological development**	1.0	20,855	5.1	132	48	277.8	232.4–327.2
F80–83	Other developmental disorders	0.6	12,067	3.0	76	26	289.9	228.4–358.8
F84, excluding F84.2–84.4	Autism spectrum disorders	0.5	9,686	2.4	61	24	256.2	195.9–324.5
**F90–98**	**Behavioural and emotional disorders with onset usually occurring in childhood and adolescence**	2.0	41,997	12.8	329	96	341.1	305.2–378.9
F90.0, F90.8, F90.9, F98.8	ADHD spectrum	1.6	34,928	10.9	281	82	344.1	305.0–385.5
F91–F92, F90.1	Conduct disorder	0.1	2,981	1.4	35	7	536.6	373.5–729.1
**F99**	**Unspecified mental disorder**	0.5	10,145	4.7	122	18	669.9	556.3–794.0

Bolded terms indicate diagnostic categories under which specific diagnoses are listed.

**Table 3.** Observed (Obs) and Expected (Exp) Number of Cases of Psychiatric Disorders and Standardized Incidence Ratios (SIR) with 95% Confidence Intervals (95% CI) Among Women with Diagnosed Gambling Disorder (GD; F63.0) in 2011–2022 (Reference Population: Women in Finnish General Population).

		Women in Finnish population (*N* = 2,231,206)		Women with GD (*N* = 1,031)				
	Diagnostic group	%	*N*	%	*N* observed	*N* expected	SIR	95% CI
**F00–99 excluding F63.0**	**Mental disorder**	42.1	938,674	92.0	949	456	208.2	195.2–221.6
**F00–09**	**Organic mental disorder**	6.3	140,839	3.8	39	13	294.8	209.5–394.5
**F10–19**	**Substance use disorder (SUD)**	3.8	84,764	30.7	317	42	761.6	680.0–847.7
F10	Alcohol use disorder (AUD)	2.6	58,885	21.7	224	28	788.6	688.7–895.3
F11	Opioid use disorders (OUD)	0.3	5,983	2.9	30	4	729.4	491.8–1013.8
F12	Cannabis use disorder (CUD)	0.1	3,041	2.4	25	2	1214.4	785.0–1737.0
F13	Benzodiazepine/hypnotics use disorder	0.4	9,641	5.9	61	5	1240.7	948.9–1571.6
F14–15	Cocaine/other stimulant use disorder	0.1	2,993	1.5	15	2	696.9	388.8–1094.2
F16, F18–19	Other or multiple drug use disorder	0.4	8,602	5.3	55	6	974.2	733.8–1248.7
F17	Tobacco use disorder	0.7	15,684	6.0	62	8	823.7	631.4–1041.5
**F20–29**	**Schizophrenia spectrum disorder**	2.4	52,448	11.3	116	23	513.4	424.2–611.0
F20	Schizophrenia	0.9	19,657	3.1	32	8	383.5	262.1–527.9
F21	Schizotypal disorder	0.1	1,747	0.8	8	1	817.3	349.0–1481.7
F22	Persistent delusional disorder	0.6	12,719	1.6	17	3	536.7	311.9–822.2
F23	Acute psychosis	0.3	7,779	1.4	14	4	337.4	183.8–537.3
F25	Schizoaffective disorders	0.3	7,443	3.4	35	4	893.8	622.2–1214.5
F28–29	Other/unspecified non-organic psychosis	1.1	23,466	6.9	71	12	569.9	445.1–710.2
**F30–39**	**Mood disorders**	17.1	381,262	76.2	786	212	371.3	345.8–397.7
F30–31	Bipolar disorders	1.5	33,295	17.7	183	20	922.9	794.0–1061.5
F32–34	Unipolar depression incl. dysthymia	16.3	363,740	72.0	742	202	366.9	341.0–393.8
F38–39	Other/unspecified mood disorder	0.7	16,499	6.3	65	10	639.6	493.6–804.6
**F40–48**	**Anxiety disorders**	23.6	526,107	69.4	716	312	229.4	212.9–246.5
F40–41	Phobic anxiety disorders	14.6	326,541	57.9	597	199	300.7	277.1–325.3
F42	Obsessive-compulsive disorder	0.8	18,116	6.6	68	12	548.3	425.7–686.4
F43–44	Adjustment and stress related disorders	9.9	220,325	32.1	331	133	249.6	223.4–277.2
F45	Somatoform disorders	4.3	95,269	6.1	63	57	109.9	84.4–138.7
**F50–59**	**Behavioural syndromes associated with psychological disturbances and physical factors**	10.8	241,545	26.7	275	124	221.7	196.3–248.7
F50	Eating disorders	0.9	19,418	3.9	40	12	324.0	231.4–432.2
F51	Non-organic sleep disorders	9.9	221,786	23.1	238	112	213.2	187.0–241.2
F52	Sexual dysfunction	0.1	1,723	0.2	2	1	173.1	16.3–496.1
**F60–69, excluding F63.0**	**Personality disorders**	2.2	49,666	26.8	276	33	839.0	742.9–940.9
**F70–79**	**Intellectual disability**	0.5	10,476	1.3	13	5	237.8	126.1–384.6
**F80–89**	**Disorders of psychological development**	0.7	15,707	2.0	21	9	230.6	142.5–339.7
F80–83	Other developmental disorders	0.5	10,239	1.8	19	5	346.7	208.3–520.1
F84, excluding F84.2–84.4	Autism spectrum disorders	0.3	5,748	0.2	2	4	52.5	5.0–150.6
**F90–98**	**Behavioural and emotional disorders with onset usually occurring in childhood and adolescence**	2.0	43,586	11.3	116	29	400.5	331.0–476.8
F90.0, F90.8, F90.9, F98.8	ADHD spectrum	1.6	34,878	9.6	99	25	399.6	324.7–482.2
F91–92, F90.1	Conduct disorder	0.1	1,706	0.6	6	1	702.0	252.6–1376.0
**F99**	**Unspecified mental disorder**	0.6	13,332	4.4	45	8	564.2	411.4–741.0

Bolded terms indicate diagnostic categories under which specific diagnoses are listed.

Of individuals with GD, 17.9% were diagnosed with adult personality disorder. Furthermore, 22.7% had a behavioural syndrome associated with psychological disturbances and physical factors, 13.6% had schizophrenia spectrum disorder, and 12.3% had behavioural and emotional disorder with an early onset. Additionally, one in ten (10.5%) had ADHD spectrum diagnoses.

### Standardized Incidence of Comorbid Psychiatric Disorders for GD and the General Population

**Figure 1.**
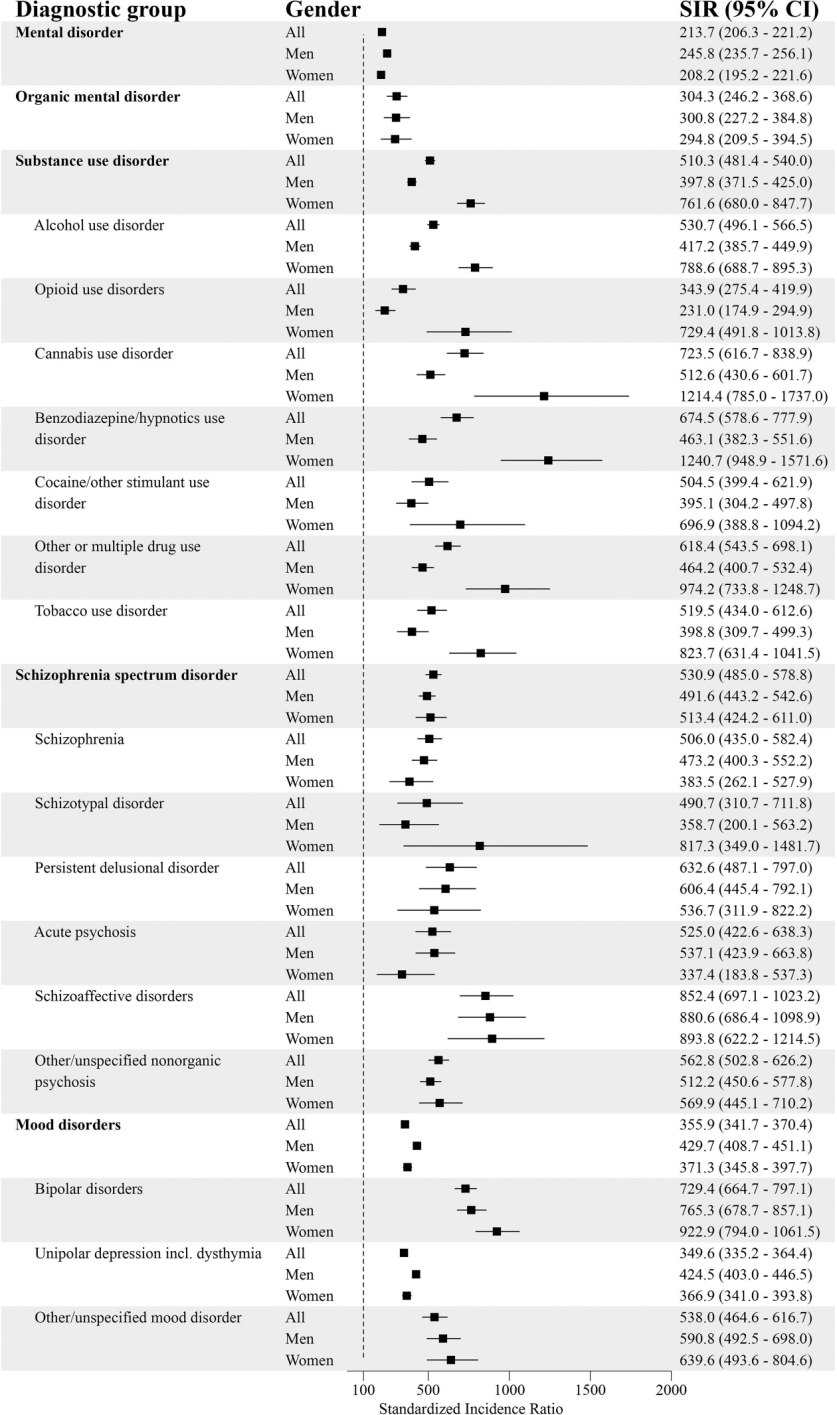
Psychiatric Disorders (Part 1) and Standardized Incidence Ratios (SIRs) With 95% Confidence Intervals (95% CI) Among People with Diagnosed Gambling Disorder (GD; F63.0) in 2011–22 (Reference Population: the Finnish General Population).

**Figure 2.**
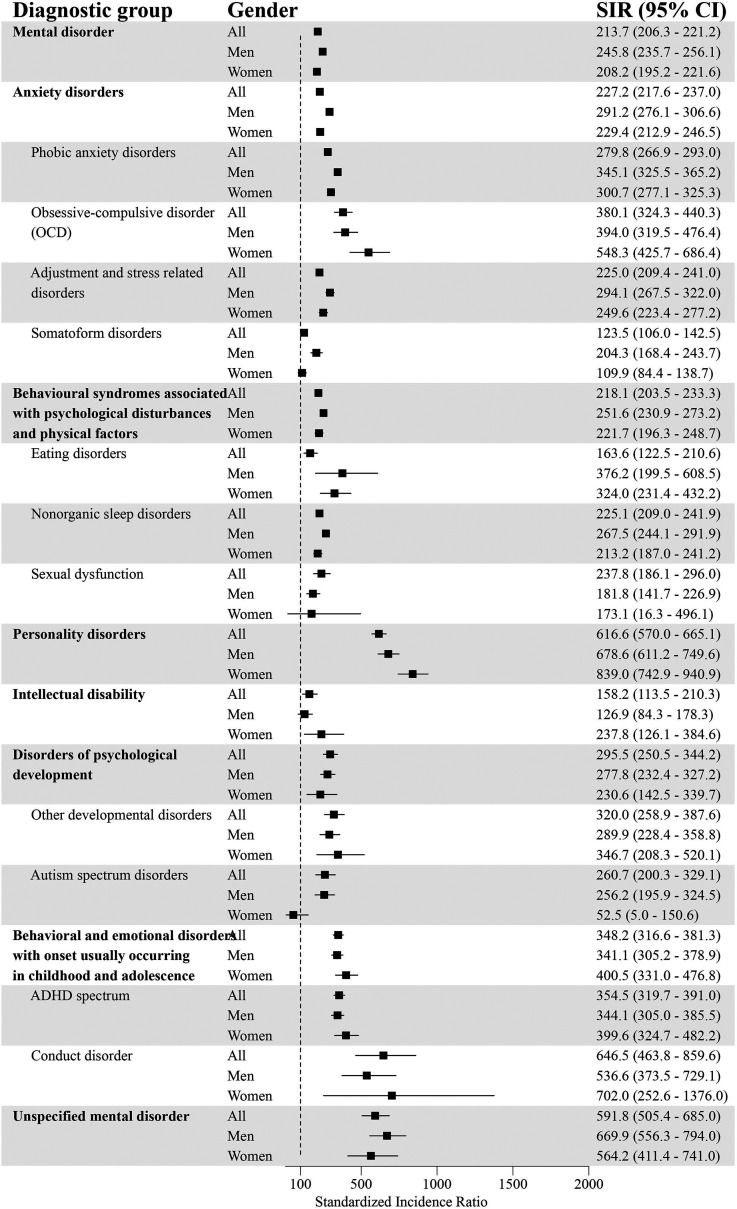
Psychiatric Disorders (Part 2) and Standardized Incidence Ratios (SIRs) With 95% Confidence Intervals (95% CI) Among People with Diagnosed Gambling Disorder (GD; F63.0) in 2011–22 (Reference Population: the Finnish General Population). ADHD = Attention-Deficit Hyperactivity Disorder.

After standardizing for age, the incidence for at least one comorbid psychiatric disorder was higher for individuals with GD compared to the general population (SIR = 213.7; 95% CI = 206.3–221.2) (Figures 1 and 2). The incidence for each diagnostic group was systematically higher in the GD population. The highest standardized incidences in different diagnostic groups were for personality disorders (SIR = 616.6; 95% CI = 570.0–665.1), unspecified mental disorder (SIR = 591.8; 95% CI = 505.4–685.0), schizophrenia spectrum disorder (SIR = 530.9; 95% CI = 485.0–578.8), SUD (SIR = 510.3; 95% CI = 481.4–540.0), and behavioural and emotional disorders with onset usually occurring in childhood and adolescence (SIR = 348.2; 95% CI = 316.6–381.3).

Among men with GD, the incidence for at least one additional psychiatric disorder was higher compared to men in the general population (SIR = 245.8; 95% CI = 235.7–256.1). The highest standardized incidences were for personality disorders (SIR = 678.6; 95% CI = 611.2–749.6), unspecified mental disorders (SIR = 669.9; 95% CI = 556.3–794.0), schizophrenia spectrum disorder (SIR = 491.6; 95% CI = 443.2–542.6), mood disorders (SIR = 429.7; 95% CI = 408.7–451.1) and SUD (SIR = 397.8; 95% CI = 371.5–425.0).

Women with GD had a higher incidence of at least one comorbid psychiatric disorder compared to women in the general population (SIR = 208.2; 95% CI = 195.2–221.6). The highest standardized incidences were for personality disorders (SIR = 839.0; 95% CI = 742.9–940.9), SUD (SIR = 761.6; 95% CI = 680.0–847.7), unspecified mental disorder (SIR = 564.2; 95% CI = 411.4–741.0), schizophrenia spectrum disorder (SIR = 513.4; 95% CI = 424.2–611.0), and behavioural and emotional disorders with onset usually occurring in childhood and adolescence (SIR = 400.5; 95% CI = 331.0–476.8).

### Deaths

Out of 3,605 individuals with GD between 2011 and 2022, there were 128 deaths (3.6%). The median age of death was 48.5 years (men = 45.5 years; women = 59.5 years) and the age at death ranged from 21 to 94 years (men = 21–85 years; women = 24–94 years). The most common causes of death were intentional self-harm by hanging, strangulation or suffocation (X70) (*n* = 11) and intentional self-poisoning (X61) (*n* = 9). Nearly one in four of these deaths (22.0%) was a suicide death. Among the suicide deaths, 68.0% were men. The median age of individuals (12 men and six women) with suicide deaths was 31.5 years (men = 31.0 years; women = 36.5 yeares) and their ages ranged from 21 to 63 years.

## Discussion

The present study examined the gender-specific SIRs of comorbid psychiatric disorders among individuals with diagnosed GD. Utilizing national healthcare registers covering the total Finnish population, the SIRs of psychiatric disorders in the GD population where compared with those in the general population. Mood and anxiety disorders were the most prevalent in both groups. However, a key finding was that PD, schizophrenia spectrum disorder and SUD were significantly more common among individuals with GD. In addition, GD was particularly associated with PD and SUD among women, and with PD and schizophrenia spectrum disorder among men. These findings contribute novel insights to the existing literature. While previous Swedish and Norwegian register-based studies on GD and comorbidities have covered both outpatient and inpatient specialized health care (Håkansson et al., 2018; Leino et al., 2023), the present study also covered primary healthcare visits.

### Psychiatric Disorders Including SUD

Of the 3,605 individuals diagnosed with GD, 88.5% had at least one additional psychiatric disorder diagnosis. The observed strong correlation between GD and other psychiatric comorbidities may indicate that diagnosed individuals are also likely to display more severe forms of the GD. The most common diagnostic categories included internalizing disorders: mood disorders and anxiety disorders. More specifically, unipolar depressive disorders/episodes and phobic anxiety disorders were the most common diagnose types. Three most prevalent comorbid psychiatric conditions were similar among men and women. Acknowledging the high rates of psychiatric comorbidities, particularly depressive symptoms that are often associated with significant financial burdens (Koomson et al., 2022; Oksanen et al., 2018; Swanton & Gainsbury, 2020) and interpersonal harms (Langham et al., 2015), it is crucial to assess the possibility of dual diagnoses (e.g., depression and gambling disorder). Suicidality is strongly linked to depressive symptoms and should be acknowledged as a significant concern. Based on another Swedish register study, women were more likely to have received another psychiatric diagnosis before being diagnosed with GD, whereas men were more likely to have received the diagnoses at the same time. However, this study and previous Finnish register studies have not examined the timing of different diagnosis (Grönroos et al., 2024; Salonen et al., 2022). Therefore, future studies are required to examine the extent to which GD precedes other health conditions and vice versa.

The incidence of all psychiatric diagnostic categories was consistently elevated in the GD population, with the highest SIRs observed for personality disorders, followed by schizophrenia spectrum disorders and substance use disorders. To our knowledge, previous research on GD and personality disorders or schizophrenia spectrum disorder seem scarce or include limited methodology (Vaddiparti & Cottler, 2017; Yakovenko et al., 2018), although the link between personality disorders and GD has been identified (Vaddiparti & Cottler, 2017). Furthermore, GD has been linked with Parkinson's disease (Heiden et al., 2017) and specifically medications increasing dopamine access in the brain (Culicetto et al., 2025; Santangelo et al., 2013).

One in ten of individuals with GD were also diagnosed with ADHD (10.5%). The incidence for ADHD was clearly higher for individuals with GD compared to the general population. Impulsivity, generally defined as acting without considering the consequences, is a characteristic of various psychiatric disorders, including ADHD, mania, and substance abuse (Diagnostic and Statistical Manual of Mental Disorders-5). Some studies have found a link between GD and ADHD (Ioannidis et al., 2019; Konstantinos et al., 2019; Lee et al., 2019; Theule et al., 2019). The Pathways Model of Problem Gambling is a widely used etiological model, which introduced three pathways for developing the problem: “behaviourally conditioned”, “emotionally vulnerable” and “antisocial impulsivists” (Blaszczynski & Nower, 2002). However, in the revised Pathways Model for Problem Gambling, impulsivity, ADHD and substance misuse were removed from Pathway 3, leaving impulsivity, antisocial traits and risk-taking behaviour in their original place (Nower et al., 2022). Despite these inconsistencies, clinicians should consider the potential connection between these two disorders, GD and ADHD, when assessing comorbidities associated with GD.

Every third (32.4%) individual with GD, also suffered from comorbid SUD, alcohol-related disorders being the most common type of SUD (24.2%). Our findings are in line with previous research indicating a clear association between GD and comorbid mental health disorders including SUD (Cowlishaw et al., 2014; Dowling et al., 2015; Håkansson et al., 2018; Leino et al., 2023). All these and other medical conditions should be considered due to their potential to increase the risk of suicidal behaviour (Østergaard et al., 2024). Additionally, it is important to evaluate the need for treatment for depression, which is strongly linked to an elevated risk of suicidal intentions, ideations and mortality (Karlsson & Håkansson, 2018).

### Suicidal Behaviour

By the end of 2022, 3.6% of individuals with GD had died. Suicide was the most common cause of death, accounting for nearly one in four fatalities (22%). Generally, individuals with GD who died by suicide tended to be relatively young, with men dying at an even younger age than women. This contradicts earlier findings, which have shown that suicidal behaviours among people with gambling problems are significantly more common among individuals older than 35 years (Armoon et al., 2023; van der Maas et al., 2024). Although, it must be noticed that in the review by Armoon et al. (2023), the relationship between age and suicide attempts was examined. The study by van der Maas et al. (2024) found a curvilinear relationship between age and gambling-related suicide, where the proportion of gambling-related cases was notably higher in the 41–50 years and 51–60 years age groups and lower in the 18–30 years and >71 years groups. Our finding highlights a concerning issue, as gambling problems are most common among younger populations, which may contribute to the increased risk of suicide in this age group. The specific characteristics and vulnerabilities of younger individuals with GD cannot be overlooked. The relatively low mean age of our sample of individual died by suicide with GD may indicate that younger individuals are particularly susceptible to the acute emotional and financial stressors associated with gambling, leading to a higher risk of suicidal behaviour. Another factor could be the influence of specific gambling behaviours and types of games popular among younger individuals, which may foster more rapid financial and emotional crises, potentially accelerating suicide risk. The rates of gambling problem seem to be most prevalent those involved in online casino or slots gambling (Tran et al., 2024).

The heightened prevalence of suicide as a cause of death among those aged 18–35 years underscores the urgent need for targeted mental health interventions and support systems for this demographic. Addressing gambling-related issues early on could potentially mitigate the risk of suicide among younger individuals, emphasizing the importance of prevention and treatment strategies tailored to their unique challenges. Future research should investigate how these age-related trends in gambling-related suicide might vary by regional and socioeconomic factors, as well as explore the roles of support networks and access to mental health resources, which could mitigate these risks among younger individuals with GD.

### Accumulation of Problems

It is noteworthy that a recently published Finnish register-based study indicated that individuals with GD not only had comorbid psychiatric disorders, but also varying comorbid somatic illness (Grönroos et al., 2024). Memory disorders, nervous system diseases, chronic respiratory diseases, diabetes and digestive diseases were particularly more common among individuals with GD compared to the general population. Moreover, individuals with GD often experience various forms of accumulated problems. A Swedish register-based study suggests that individuals with GD are at significant risk of work disability both before GD is diagnosed and for a prolonged period thereafter (Månsson et al., 2024). In addition, a Norwegian longitudinal register-based case–control study suggests that income is a risk marker for GD (Girard et al., 2023): Expected income for individuals with GD, particularly for women and for the youngest, was significantly lower compared to the average income among the general population in 2008–2018. Indeed, individuals with GD were more likely to have income levels in the bottom quartile. This finding is in line with a systematic review indicating that there is a health inequality in gambling related harms (Raybould et al., 2021). Furthermore, socially disadvantaged individuals, for example individuals living in poverty have a higher change to develop GD than the general population (Sharman et al., 2019). In the present study, we examined the SIRs of comorbid psychiatric disorders among individuals with GD. In future studies, it would be important to include socio-economic factors in the analysis by using individual-level register data, as GD and psychiatric comorbidity may be clustered within certain lower socio-economic groups. Assessing the overall situation of the individual in the treatment of GD is essential to ensure that care is fully integrated with the patient's circumstances.

### Implications for Research and Practice

Previous studies indicate that there is no one path for developing GD (Sharman et al., 2019). Instead, different demographic, social, developmental and environmental factors may interact with each other and influence one's relationship with gambling behaviour (Abbott et al., 2018; Allami et al., 2021; Dowling et al., 2017; Karlsson et al., 2021; Raybould et al., 2021; Sharman et al., 2019; Tran et al., 2024). In summary, there is still a significant paucity of research on the progression of health issues among individuals with GD. In future studies, it would be important to utilize individual-level register data to examine more closely the different trajectories of both somatic and psychiatric health issues among different subgroups of individuals with GD. The heterogeneity observed across the income distribution for patients with GD, coupled with identifiable patient characteristics for income group trajectories, may help in both the prediction and screening of GD (Girard et al., 2023).

These results highlight the need for systematic screening of GD especially in vulnerable populations and individuals seeking treatment for psychiatric symptoms, such as insomnia or anxiety. GD is rarely the primary reason for seeking treatment, although it may be a significant underlying problem. There are models and tools for early interventions, which are under-utilized in health care as well as social services (available in Finland: Gambling problem (rahapeliongelma): Current Care Guidelines, 2023). Systematic evaluation of suicidal ideation and suicide risk should be a part of a comprehensive evaluation of GD. Treatment of comorbid psychiatric disorders is crucial for successful treatment of GD and vice versa: untreated GD may worsen the prognosis of comorbid psychiatric disorders, such as depression.

Importantly, although the administrative prevalence rates of GD have increased in Finland (Salonen et al., 2022), the observed rates are extremely low in Finland and other Nordic countries (Håkansson et al., 2018; Latvala et al., 2023; Salonen et al., 2022). The annual prevalence of diagnosed GD increased in Finland from 4.6 cases per 100,000 inhabitants to 21.4 cases per 100,000 inhabitants between 2011 and 2021 (Latvala et al., 2023). Such data imply that the problem remains under-diagnosed: it is likely that many individuals suffering from GD are neither seeking medical treatment nor being identified during doctors’ visits. In Finland, a GD diagnosis is always set by a medical doctor, but other social and healthcare professionals may use the diagnostic codes as a part of their own documentation. The treatment of GD is legally the responsibility of public health care and primarily funded through taxation. While some private clinics offer GD treatment, they are not the main providers. Private healthcare providers were integrated into these registers in 2019. However, many private organizations and NGOs (non-governmental organizations), such as psychotherapy providers, helplines and peer support groups, do not systematically collect and/or report diagnostic data, as they often operate outside the public healthcare system. Since 2023, legislation has clarified that addiction treatment is part of health care, while addiction-related social work remains within social services, which do not use diagnostic codes. This structural division may contribute to underreporting and selection bias in register-based data. Thus, although private and third-sector services contribute to GD treatment, the majority of diagnostic data originate from public healthcare settings. Furthermore, even if the individual has been diagnosed with GD, our results do not indicate whether they were offered or received any treatment/help particularly for GD. Further studies evaluating psychosocial outpatient and inpatient treatment linked with GD are needed. On the other hand, it is possible that they might have received help or support from social and healthcare professionals without a GD diagnosis.

### Strengths and Limitations

Internationally, epidemiological studies on problem gambling have largely relied on population-based survey samples (Finnish Institute for Health and Welfare, 2024; Tran et al., 2024; Gabellini et al., 2023; Calado & Griffiths, 2016). High-quality data based on administrative health registries bring an important insight to most severe gambling problems. Both GD and comorbid diagnoses were evaluated only among adults and during a limited period: between years 2011 and 2022. The strength of this study is that all figures do represent the total adult population in Finland and cover both primary and specialized care, while prior studies are based on specialized healthcare registers only. However, it should be noted that, since 2020 in Finland, private service providers and occupational healthcare providers have contributed data to the primary care register more comprehensively than before. By 2022, the coverage of occupational health care is considered fairly comprehensive, although some provider-specific gaps still remain. In Sweden and Finland, the rates of diagnosed GD have increased across gender and age groups during the past decade (Håkansson et al., 2024; Salonen et al., 2022). Although our aggregated data cover the entire adult population in Finland, it should be acknowledged that health seeking individuals with GD diagnoses have a median age ranging 33.5–36.0 years (Salonen et al., 2022). It should be noted that any conclusions on trajectories of the problems cannot be drawn since our results do not tell which diagnose was first: GD or the comorbid occurring one. Lastly, concerns that GD diagnose may also be used in the clinical practice for symptoms associated with gaming disorder ought to be acknowledged (Karlsson & Håkansson, 2018; Salonen et al., 2022). It should also be noted that individuals with diagnosed GD are likely to be those who use healthcare services more frequently, which may partly explain the higher observed prevalence of other mental health diagnoses compared to the general population. This may introduce a selection bias toward more severe or help-seeking cases. Furthermore, in our analysis, we cannot calculate precise mortality rates due to the use of aggregated data. Also, we were unable to calculate SIRs, as our data request did not include the number of suicide deaths in the general population. We agree that future research should address these gaps to provide a clearer understanding of suicide risk in this population.

## Conclusions

Increased psychiatric comorbidity, mortality and suicidality compared to the general population, were clearly linked to clinically diagnosed and recorded gambling disorder (GD). Among individuals diagnosed with GD, a substantial majority (88.5%) also met the diagnostic criteria for at least one other psychiatric disorder. The most prevalent comorbidities were mood and anxiety disorders. However, personality disorders, schizophrenia spectrum disorder and SUD were significantly more common among individuals with GD compared to those in the general population. In addition, GD was particularly associated with personality disorders and SUD among women, and with personality disorders and schizophrenia spectrum disorder among men. Our results underscore the need for social and healthcare professionals to be aware of the potential presence of comorbid psychiatric conditions and the high risk of suicide when working with individuals with GD and vice versa. In the treatment of GD, assessing the overall situation is essential to ensure that care is fully integrated with the patient's circumstances. Raising awareness among health and social workers regarding the connection between GD and psychiatric comorbidities is vital for both detecting problems early and enhancing the treatment planning process.
